# Supplementary material for: Studying the Influence of Salt Concentrations on Betalain and Selected Physical and Chemical Properties in the Lactic Acid Fermentation Process of Red Beetroot
Source: Molecules. 2024 Oct 11;29(20):4803. doi: 10.3390/molecules29204803 (PMC11510701; doi:10.3390/molecules29204803)
Supplement: Supplementary file 1 [file molecules-29-04803-s001.zip › molecules-3203669-supplementary.pdf]

Studying the influence of salt concentrations on betalain and selected physical and chemical properties in the lactic acid fermentation process of red beetroot

Emilia Janiszewska-Turak [1,*], Anna Wierzbicka[1], Katarzyna Rybak[1], Katarzyna Pobiega[2], Alicja Synowiec[2], Łukasz Woźniak[3], Urszula Trych[4], Andrzej Krzykowski[5] and Anna Gramza-Michałowska[6]

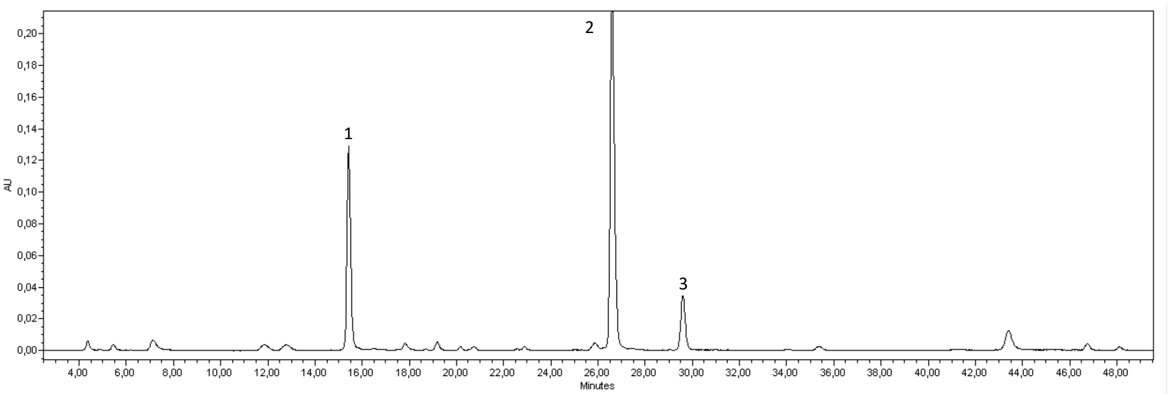

Figure S1. Chromatogram showing HPLC analysis of betaxanthins in raw beetroot (fresh) at 480 nm. 1- vulgaxanthin I (RT= 15 min), 2- betanin (RT=27 min), 3- isobetanin (RT=29 min), and other small peaks from 10 to 2 min were quantified together and described as "other betaxanthins".

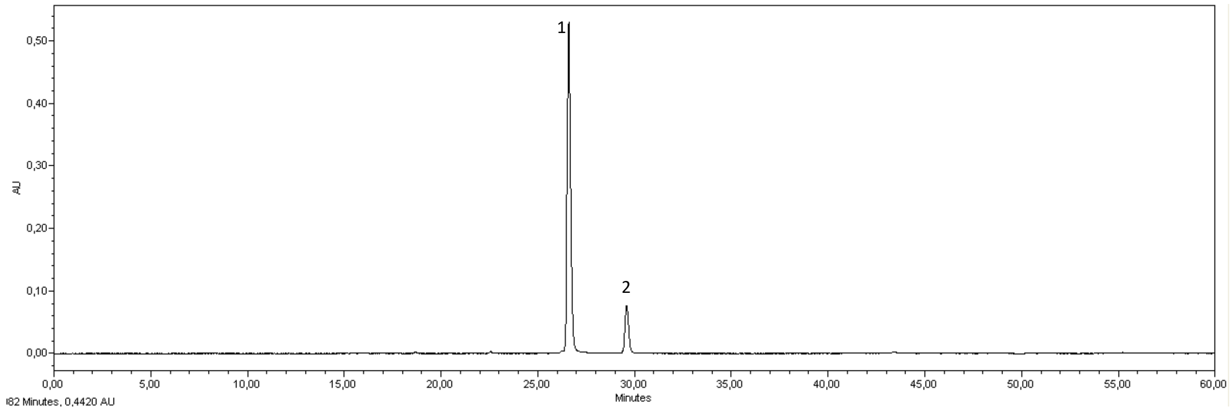

Figure S2. Chromatogram showing HPLC analysis of betacyanins in raw beetroot (fresh) at 538 nm. 1-betanin (RT=27 min), 2-isobetanin (RT-29 min).
